# P-2057. Immunogenicity of COVID-19 Booster Vaccines in Mexico and Mongolia

**DOI:** 10.1093/ofid/ofae631.2213

**Published:** 2025-01-29

**Authors:** Justino Regalado-Pineda, Paola del Carmen Guerra-de-Blas, Megan C Grieco, Munguntsetseg Batkhuu, Eduardo Becerril-Vargas, Naranjargal Dashdorj, Dehkontee Dennis, Helene Highbarger, Sally Hunsberger, Ganbolor Jargalsaikhan, Selwrenee Mapleh, Ashley McCormack, Garmai Nyuangar, Juan Pablo Ramírez-Hinojosa, Renee Ridzon, Yuri Alfonso Roldán-Aragón, Guillermo Miguel Ruiz-Palacios, Jesús Sepúlveda-Delgado, Irini Sereti, Katy Shaw-Saliba, Mary Smolskis

**Affiliations:** Instituto Nacional de Enfermedades Respiratorias Ismael Cosío Villegas, Mexico City, Distrito Federal, Mexico; The Mexican Emerging Infectious Diseases Clinical Research Network (LaRed), Mexico, Distrito Federal, Mexico; National Institute of Allergy and Infectious Diseases, Bethesda, Maryland; Onom Foundation, Ulaanbaatar, Ulaanbaatar, Mongolia; Instituto Nacional de Enfermedades Respiratorias Ismael Cosío Villegas, Mexico City, Distrito Federal, Mexico; Onom Foundation, Ulaanbaatar, Ulaanbaatar, Mongolia; Partnership for Research on Vaccines and Infectious Diseases in Liberia, Monrovia, Montserrado, Liberia; Frederick National Laboratory, Rockville, Maryland; National Institute of Allergy and Infectious Diseases, Bethesda, Maryland; Onom Foundation, Ulaanbaatar, Ulaanbaatar, Mongolia; Partnership for Research on Vaccines and Infectious Diseases in Liberia, Monrovia, Montserrado, Liberia; Frederick National Laboratory for Cancer Research, Frederick, Maryland; Partnership for Research on Vaccines and Infectious Diseases in Liberia, Monrovia, Montserrado, Liberia; Hospital General Dr. Manuel Gea Gonzalez, Mexico City, Distrito Federal, Mexico; National Institute of Allergy and Infectious Diseases, Bethesda, Maryland; Hospital General Dr. Aurelio Valdivieso, Oaxaca, Oaxaca, Mexico; Instituto Nacional de Ciencias Médicas y Nutrición Salvador Zubirán, Mexico, Distrito Federal, Mexico; Hospital Regional de Alta Especialidad Ciudad Salud, Tapachula, Chiapas, Mexico; Laboratory of Immunoregulation, NIAID, Bethesda, MD; The National Institute of Allergy and Infectious Diseases, National Institutes of Health, Bethesda, Maryland; National Institute of Allergy and Infectious Diseases, Bethesda, Maryland

## Abstract

**Background:**

InVITE (NCT# 05096091) is a 7-country observational study assessing immune responses to COVID-19 vaccines offered from national vaccination programs. This analysis focuses on the response 2 months after receipt of a booster vaccine (third dose) among participants from Mexico and Mongolia. Vaccines assessed were mRNA (Comirnaty, Pfizer (BioNTech), non-replicating viral vector (Vaxzevria, Oxford/AstraZeneca; Sputnik-V, Gam-COVID-Vac), or inactivated virus vaccines (CoronaVac, SinoVac and Covilo, Sinopharm).

**Figure:**
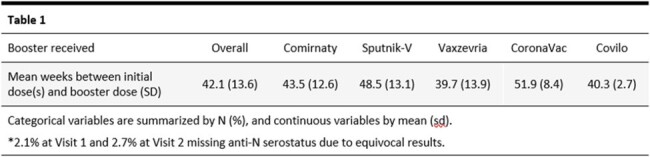

**Methods:**

Blood samples were taken at baseline (Visit 1) and 2 months after receipt of booster (Visit 2). Information on primary vaccine regimens (receipt of first 2 COVID-19 vaccine doses) was collected. Quanterix SARS-CoV-2 Spike IgG assay measured anti-Spike (S) antibody levels. BioRad Platelia SARS-CoV-2 assessed anti-Nucleocapsid pan Ig (anti-N) antibody levels. Multivariate regression models were used to compare booster vaccines and regimens (primary+booster) based on log10 anti-S levels. CoronaVac and Covilo were excluded from some analyses due to small numbers (≤10 participants).

**Figure:**
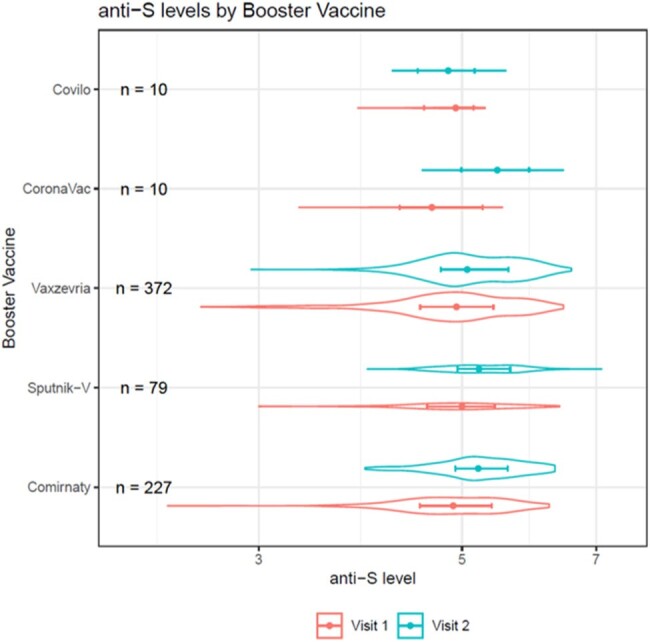

Violin plot with estimates and 95% confidence intervals (CI) of anti-S levels at Visit 1 and Visit 2. Groups with >10 participants were included.

**Results:**

We report on 698 participants with Visit 1 and Visit 2 anti-S levels measured. 59.5% were women, 46.3% were 40-59 and 24.4% were ≥60 years old. 33.7% reported hypertension/diabetes and 22.2% had body mass index >30. 65.6% and 70.8% were positive for anti-N antibodies, at Visit 1 and 2, respectively. Anti-S levels at Visits 1 and 2 are shown in Figure 1. After adjusting for country, the booster with Comirnaty produced higher anti-S levels compared to Vaxzevria and Sputnik V, and Sputnik V produced relatively higher levels than Vaxzevria (Figure 2). When comparing combinations of primary and booster vaccines, Comirnaty + Comirnaty had the highest anti-S levels (Figure 3).

**Figure:**
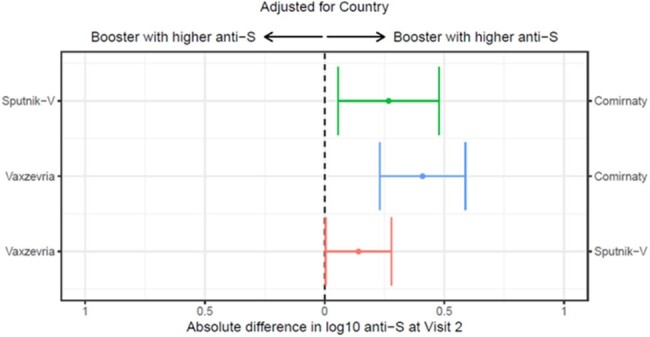

Difference between log10 anti-S levels at Visits 1 and 2 with 95% CI for comparison of booster vaccines, adjusted for country.

**Conclusion:**

Immune response to booster COVID-19 immunization differed by vaccine with the highest response seen when Comirnaty was included in a regimen. These data, including vaccines and geographic locations rarely reported in other studies, enrich understanding of immunogenicity to different combinations of vaccines used as primary and booster doses.

Figure 3

**Figure:**
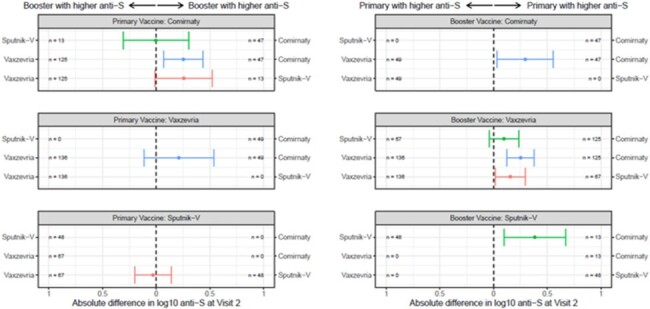

Difference between vaccine combinations (primary vaccine + booster vaccine) log 10 anti-S levels with 95% CI to compare combinations. Rows without a line indicate 'no data' for those combinations.

**Disclosures:**

Juan Pablo Ramírez-Hinojosa, MD, Abott: Honoraria|Jansen: Honoraria|Silanes: Honoraria Irini Sereti, M.D., NeoImmunotech: Collaboration

